# The Nature of Attachment Relationships and Grief Responses in Older Adults: An Attachment Path Model of Grief

**DOI:** 10.1371/journal.pone.0133703

**Published:** 2015-10-13

**Authors:** Yan Kho, Robert T. Kane, Lynn Priddis, Josephine Hudson

**Affiliations:** School of Psychology and Speech Pathology, Curtin University, Bentley, Western Australia, Australia; Harvard Medical School, UNITED STATES

## Abstract

**Background:**

Various researchers have theorized that bereaved adults who report non-secure attachment are at higher risk of pathological grief. Yet past findings on avoidant attachment representations and grief have yielded limited and contradictory outcomes. Little research has been conducted with older adults to identify the psychological processes that mediate between self-reported attachment representations and the patterns of grief.

**Objective:**

To examine the impacts of avoidant attachment and anxious attachment dimensions on emotion and non-acceptance, in response to the loss of a conjugal partner, and the mediating effect of yearning thoughts.

**Design:**

Men (*N* = 21) and women (*N* = 68) aged 60 years and above who had lost a partner within the last 12 to 72 months were invited to participate. Participants rated their levels of yearning thoughts about the deceased, emotions and non-acceptance on the Texas Revised Inventory of Grief (TRIG-Present), and their type and level of general romantic attachment on the Experiences In Close Relationship questionnaire (ECR).

**Results:**

Structural equation modelling (SEM) indicated that individuals who reported higher levels of avoidant attachment reported less emotional responses and less non-acceptance. SEM also showed that individuals who reported higher levels of anxious attachment reported greater emotional responses and greater non-acceptance. SEM further indicated that these relationships were mediated by yearning thoughts.

**Conclusion:**

People adopt different grief coping patterns according to their self-reported attachment representations, with the nature of their yearning thoughts influencing the process. Grief therapy may be organized according to individual differences in attachment representations.

## Introduction

In 1990, 11% of the Australian population were aged 65 years or older. This figure rose to 14% in 2010 [1]. With the higher risk of accumulative bereavement in late life [2], and an aging population, individual differences in the grieving needs of bereaved older adults seek to be further addressed.

Researchers, beginning with Bowlby [3], suggest that individuals’ attachment representation significantly influences their process, patterns and outcome of affect regulation, such as grieving. Attachment representations are cognitive-affective structures based on generalized memories of the sensitivity and consistency of the responses of one’s earliest attachment figures, usually the first and primary caregivers, towards one’s proximity-seeking attempts for psychological security [3]. Due to the repeated nature of these earliest attachment experiences, the memories are internalized to provide expectations about how significant others are likely to provide support and comfort during stressful times in attachment relationships, the most extreme being the death of one’s partner [3].

Attachment representations have been conceptualized as degrees of avoidant attachment and anxious attachment. Adults who self-report as higher on avoidant attachment in romantic relationships typically strive for independence and emotional distance from their partner [4, 5]. Some researchers further construe the avoidant attachment continuum as either dismissing-avoidant wherein one has little *conscious* regard for interpersonal closeness or as fearful-avoidant whereby one prefers autonomy out of fear of being let down in relationships [6]. A second dimension in this model is that of anxious attachment. Adults who self-report as higher on anxious attachment are excessively dependent on the partner and worry that he/she will not be available and supportive in times of need [4, 7]. Based on the two-dimensional (avoidant-anxious) model of attachment, adults who self-report as low on both avoidant and anxious attachment dimensions are considered to be the prototypical securely-attached individuals [6]. Securely attached adults are characteristically more comfortable with emotional closeness and also more confident about their partners’ emotional responsiveness [4].

Several studies indicate that avoidantly attached adults and anxiously attached adults vary in the nature and treatment of their attachment-related thoughts, leading to differences in how they cope with and express their distress over an attachment loss, such as the death of a loved one. Relative to other adults, avoidantly attached adults are less attentive to attachment-related concerns [8, 9, 10], and suppress attachment-related thoughts more successfully [4, 5, 7, 11]. These cognitive defenses evidence what Bowlby has originally termed as defensive exclusion, which he describes as avoidantly attached adults’ maladaptive way of avoiding attachment-related distress [3]. Tests (e.g., Stroop colour or lexicon decision tasks), interviews and brain scans on *implicit* cognitive processing revealed that beneath their *minimalist* concern with attachment needs, avoidantly attached adults indeed experience heightened yearning thoughts for their attachment figures during attachment threats but usually only when under *high* cognitive load imposed by having to simultaneously process multiple stimuli [4, 11]. They also experience emotional distress [12]. Bereaved avoidantly attached adults are also at higher risk of somatic symptoms [13]. These psycho-cognitive/psycho-neurological findings appear to provide evidence against stipulations [7, 14] that dismissing-avoidantly attached adults are more emotionally resilient than fearful-avoidantly attached adults. Conversely, anxiously attached adults demonstrate *both implicit and explicit* cognitive hypervigilance to attachment-related negative memories, cues and threats, and higher attachment-related distress [4, 5, 10, 12].

The existing data on the differential patterns of attachment-related thoughts and affect regulation among avoidantly attached adults and anxiously attached adults begs the question of whether and how bereaved older adults may grieve differently due to their varying patterns of attachment. While past studies have either found contradictory or no links between avoidant attachment representations and grief, they have unequivocally posited that anxiously attached bereaved adults experience greater grief [13, 14, 15, 16]. Conversely, bereaved securely attached adults tend to experience less grief over time [14]. Previous attachment-grief studies [13, 14, 15, 16] have all conceptualized grief as a unitary construct, despite strong evidence that the construct of grief is complex and comprises a number of domains. Grief domains include preoccupied, yearning thoughts for the deceased [14, 17, 18, 19] emotions of sadness [14, 17, 18, 19], and the degree of acceptance/non-acceptance about the loss [14, 18, 19]. Grief, when measured multi-dimensionally, allows for a richer investigation into the specific role of individual grief factors in the grieving process and outcome [20]. Measuring grief unidimensionally may have therefore limited findings on any existing association between the avoidant attachment representation and grief, and thus the understanding of the role of yearning thoughts in the process of grieving. Older adults are also more likely than younger adults to self-report as avoidantly attached, especially as dismissing-avoidantly attached [21, 22], possibly in adjustment to age-related losses [2]. Yet no study had been conducted exclusively with older adults.

**Fig 1:**
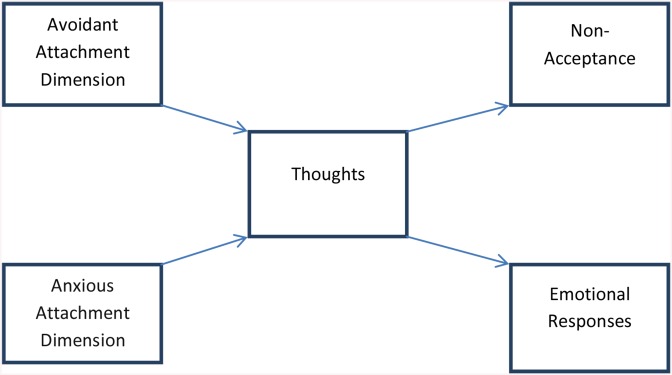
A full mediator model of grief.

The current study aimed to test a mediator model of grief on a sample of older adults. The *full* mediator model is illustrated in Fig 1. The model yielded two primary hypotheses:

H1: Individuals with *higher levels* of self-reported romantic avoidant attachment will report *fewer* yearning thoughts about the deceased, leading to self-reports of *lower* levels of emotional responses and *lower* levels of non-acceptance about the loss.
H2: Individuals with *higher* levels of self-reported romantic anxious attachment will report *more* yearning thoughts about the deceased, leading to self-reports of *higher* levels of emotional responses and *higher* levels of non-acceptance.

## Method

### Participants

Participants were recruited from senior centres and newspapers. The inclusion criteria were: Aged sixty years or older, the death of a spouse/long-term partner within the last 1 to 6 years, a relationship with the deceased for five years or more prior to the bereavement, English as a first/native language or possess sufficient English proficiency to read a standard English newspaper, and not presently suffering from depression or dementia. One hundred and twelve individuals who are residents of Australia volunteered to participate in the study. Three did not complete both questionnaires, nineteen did not return the questionnaires, and one had been bereaved for more than 6 years and was excluded from the study.

The final sample comprised 89 older adults, ranging from 60 to 92 years (*M* = 73.94 years, *SD* = 8.36). 61 (69%) are women and 28 are (31%) men. Of the 73 participants who had specified the length of their relationship with the deceased, the relationship ranged from 5 to 64 years (*M* = 42.23 years, *SD* = 13.56). The remaining 16 participants had self-reported that the duration of their relationship had been at least 5 years but did not specify the exact duration. Of the 76 participants who had specified time since loss, time ranged from 12 to 72 months (*M* = 39.87 months, *SD* = 20.75). The remaining 13 participants had self-reported that they had been bereaved for a minimum of 12 months but not more than 72 months, without indicating the exact length of time.

### Measures

#### Romantic attachment dimensions

Experiences in Close Relationship (ECR) [23] is a 36-item self-report questionnaire that assesses the participants’ levels of avoidant attachment and anxious attachment. Eighteen items relate to avoidant attachment (e.g., “I worry about my partners getting too close to me”). Eighteen items pertain to anxious attachment (e.g., “I want to merge completely with my partner”). Items are answered on a 7-point Likert scale.

Each participant rated how well each of the thirty-six statements described his/her feelings towards intimate partners in general. Akin to past reviews on the ECR [24], the ECR demonstrated good internal reliability in the present study, with Cronbach’s alpha coefficients of .88 for avoidant attachment dimension and .81 for anxious attachment dimension.

#### Grief dimensions

The Texas Revised Inventory of Grief-Present scale (TRIG-Present) [25] is a 13-item self-report questionnaire that quantifies current grief for a deceased person. In the original factor analysis by Futterman and colleagues, the 3-factor model of grief (Thoughts, Emotions and Non-Acceptance) fits better than the single-factor model [20]. Thoughts consists of five items which assess participants’ level of yearning thoughts about the dead partner (e.g., “Sometimes I very much miss my spouse/long-term partner who died”). Emotions contains five items which measure participants’ degree of emotional distress (e.g., “I still cry when I think of my spouse/long-term partner who died”). Non-Acceptance comprises three items which measure participants’ degree of non-acceptance about the loss (e.g., “I cannot accept my spouse/long-term partner’s death”). Items are answered on a 5-point Likert scale. Participants were instructed to reflect on their level grief for the past week in these three domains. Similar to the high internal consistency demonstrated originally by the 3-factor TRIG-Present [20], the current data yielded Cronbach’s alphas of .83 for Thoughts, .86 for Emotions and .77 for Non-Acceptance.

### Procedure

Ethics approval was granted by the Curtin University Human Research Ethics Committee. Written informed consent for participation in the study and for publication of de-identified group data were obtained from the participants. Each participant was provided with an explanatory letter, consent form, background information checklist, TRIG-Present form and ECR form. These were returned to the researchers by email, mail or in person. The researchers also carried out follow-ups through phone calls three days after receiving the completed questionnaires, to ensure participants’ emotional well-being. Coordinators for each of the centres were provided with information sheets about behavioural symptoms of re-activated grief and free counselling helplines.

### Statistical Analysis

Structural equation modelling (SEM) was conducted using LISREL software (Version 8.54). SEM is used to test the viability of causal models. Unlike traditional path models, which only incorporate observed variables measured with error, SEMs include both observed and latent variables. By including both observed and latent variables in the modeling process, SEM is able to account for measurement error in the observed variables [26].

**Fig 2:**
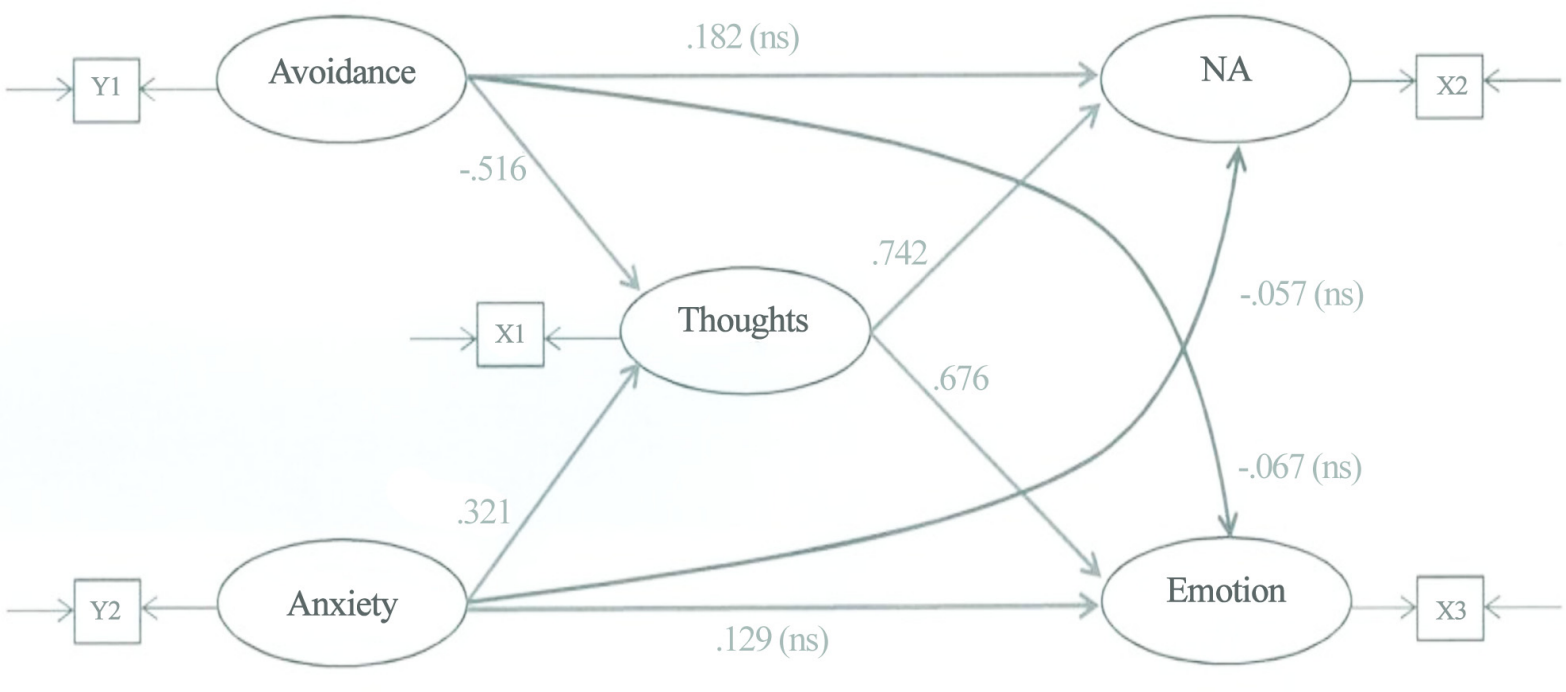
The saturated model (all possible left-to-right pathways). ECR—Avoidant Attachment scale (Y1); ECR—Anxious Attachment scale (Y2); TRIG-Present—Thoughts scale (X1); TRIG-Present—Non-Acceptance scale (X2); TRIG-Present—Emotions scale (X3).

**Fig 3:**
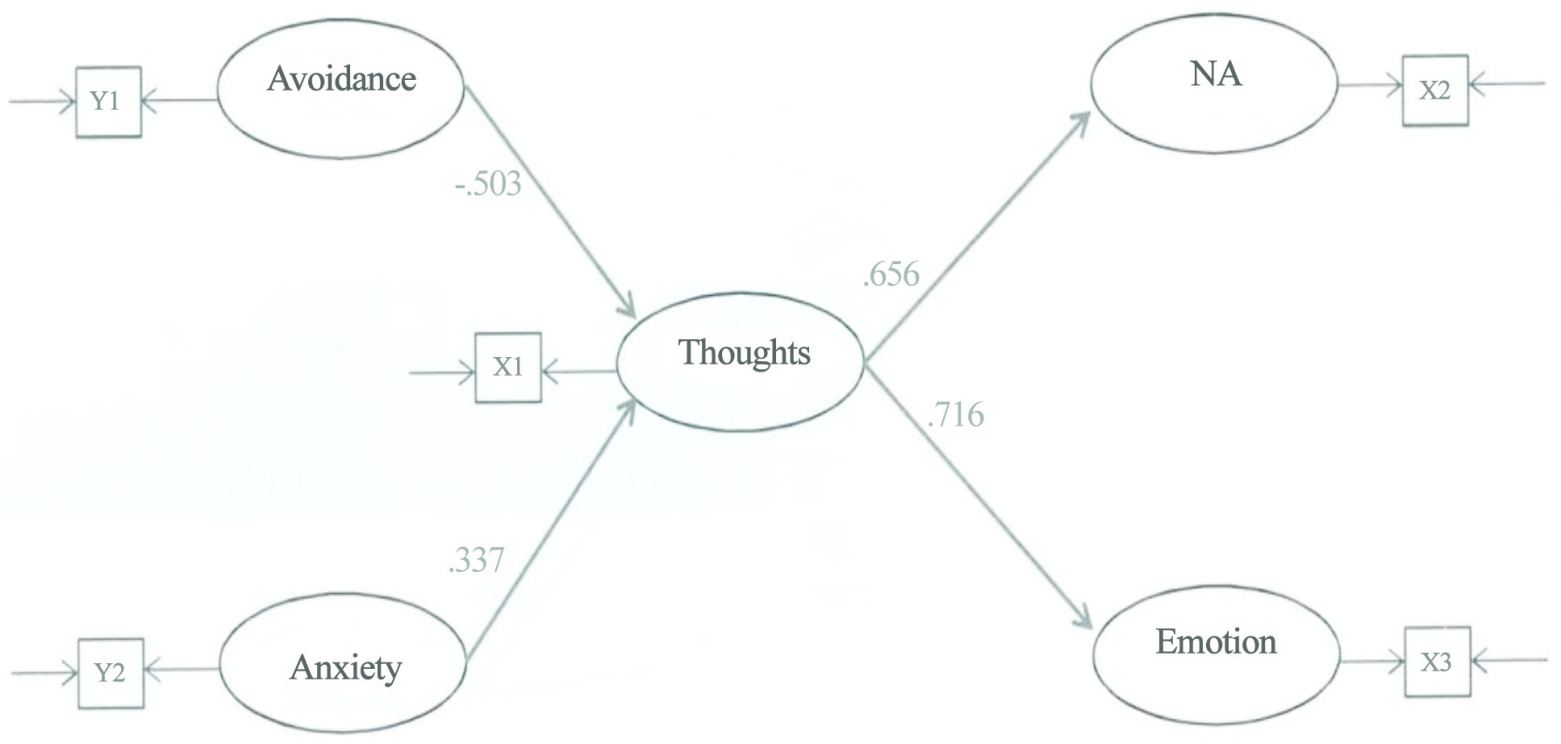
The mediator model (direct pathways were non-significant and therefore they were deleted from the model). ECR—Avoidant Attachment scale (Y1); ECR—Anxious Attachment scale (Y2); TRIG-Present—Thoughts scale (X1); TRIG-Present—Non-Acceptance scale (X2); TRIG-Present—Emotions scale (X3).

The basic analytical strategy involved comparing a saturated model (Fig 2) to a complete mediator model (Fig 3) in order to determine which provided a better fit for the data. The saturated model will fit the data better than the mediator model simply because it has more pathways; but the question is: Does the saturated model fit the data significantly better than the mediator model? If it does, then the saturated model (which would be inconsistent with H1 and H2) would be opted for in favour of the mediator model. If there is no significant difference between the two fits, then the more parsimonious mediator model is opted for (which would be consistent with H1 and H2). The SEM accounts for measurement error by including both latent variables (represented by the ovals) and observed variables (represented by the squares). Figs 2 and 3 indicate that each latent construct is measured by just one observed variable. In these circumstances, the measurement error associated with each observed variable is fixed to one minus the reliability coefficient of the variable and its factor loading is fixed to the square root of its reliability coefficient [27]. These fixed parameters are input to LISREL prior to SEM. The more complex saturated model has 15 free parameters that must be estimated from the data. In order to reliably test the measurement model, it has been recommended that there are at least 5 participants for each free parameter in the measurement model [26]. An adequate sample size for testing the saturated model would therefore be 75 (= 5 x 15).

## Results

The following fit indices were used to evaluate model fit: the Comparative Fit Index (CFI), the Non-Normed Fit Index (NNFI), the Standardised Root Mean Square Residual (SRMR), and the Root Mean Square Error of Approximation (RMSEA). The fit statistics for the two models are reported in Table 1.

**Table 1:** Goodness-of-fit Indicators for the Mediation Models (*N* = 89).

Structural models	*χ* ^2^	*df*	*χ* ^2^/*df*	CFI	NNFI	SRMR	RMSEA (CI)	Model AIC
Saturated	4.66	1	4.657	.963	.628	.032	.204 (.0501, .405)	32.66
Mediator	7.35	5	1.47	.972	.944	.045	.073 (.000, .177)	27.35

Individual participants’ mean scores for Avoidant Attachment, Anxious Attachment, Thoughts, Emotions, and Non-Acceptance are reported in S1 Fig.

Fit statistics for the two models are reported in Table 1.

The χ^2^/df, CFI, NNFI, and RMSEA favour the mediator model (χ^2^/df < 3 [26], CFI > .95 [28], NNFI > .94 [28], and RMSEA = .073 with a 90% confidence interval that includes .05 [26]. The mediator model also has a smaller Model AIC indicating a better fit. In contrast, the SRMR fit both models (SRMR < .1) but favoured the saturated model. The *χ* ^2^ difference test, however, was non-significant (*χ* ^2^_diff_ = 2.69, *df* = 4, *p* = .625) indicating that the *χ* ^2^ value for the saturated model was not significantly smaller than the *χ* ^2^ value for the mediator model. The weight of the evidence indicates that the mediator model is at least as good as the saturated model at explaining the data. Scientific parsimony therefore dictates that we opt for the less complex mediator model.

The *p*-values for the indirect pathways in the mediator model were subsequently examined to confirm that Thoughts was mediating the relationships between the exogenous variables (Avoidance, Anxiety) and the endogenous variables (NA and Emotion). An initial requirement that must be met in order for Thoughts to be a mediator is the significance of each of the four *component* pathways that comprise the mediation effects. Fig 2 indicates that these pathways are significant. A second requirement is the significance of the *overall* indirect effects. The strength of each of the four indirect effects is given by the product of its two component path coefficients. These effects are reported in Table 2.

**Table 2:** Indirect Effects for the Mediator Model (*N* = 89).

Indirect pathways	*First Component*	*Second component*	*Overall effect (1* ^*st*^ *x 2* ^*nd*^ *component)*	*p*-value
Avoidance →Thought→ NA	-.503	.656	-.330	< .001
Avoidance →Thought→ Emotion	-.503	.716	-.360	< .001
Anxiety →Thought→ NA	.337	.656	.221	.015
Anxiety →Thought→ Emotion	.337	.716	.241	.013

All indirect effects are significant.

## Discussion

### Summary of results

Each of the four pathways in the mediator model was significant. The relative directions of the path coefficients (positive or negative) indicated that individuals with *higher* levels of self-reported romantic avoidant attachment tended to report *fewer* yearning thoughts about the deceased, and individuals who reported *fewer* yearning thoughts tended to report *lower* levels of emotional responses and *lower* levels of non-acceptance about the loss. H1 is therefore supported. The path coefficients also indicated that individuals with *higher* levels of self-reported romantic anxious attachment tended to report *more* yearning thoughts about the deceased, and individuals who reported *more* yearning thoughts tended to report *higher* levels of emotional responses and *higher* levels of non-acceptance about the loss. H2 is therefore supported.

### Romantic Avoidant Attachment Dimension and Grief

This study examined the impact of older adults’ self-reported non-secure attachment representations on their yearning thoughts, emotions and non-acceptance in response to conjugal bereavement. The result supported the hypothesis that participants who self-report higher scores on the ECR avoidant attachment dimension would experience less emotional distress and less non-acceptance, modulated by their fewer yearning thoughts about the deceased, as self-reported by the TRIG-Present.

This is a first study that empirically demonstrated the cognitive link between individuals’ avoidant attachment representation and grief. Consistent with the position that avoidantly attached adults tend to minimize attachment-related thoughts and that a reduction in these thoughts leads to less emotional distress [7, 13, 15], the mediator pathways were significant (as shown in Figs 2 and 3), while the direct pathways between participants’ avoidant attachment dimension and their levels of emotions and non-acceptance were non-significant (as indicated in Fig 2). The negative relationship between participants’ avoidant attachment dimension and their degree of yearning thoughts is in keeping with past literature that avoidantly attached adults [4, 5, 8, 9, 11] and dismissing-avoidantly attached adults [7] are cognitively defensive against attachment-related concerns.

While the avoidant attachment mediator pathways might have reflected the impact of older adults’ *dismissing-avoidant* attachment representation (see reviews in [7, 9, 21, 22]), these pathways might, more plausibly, echo the cognitive and emotional defensiveness of *both* avoidant subtypes. The cognitive defenses of avoidantly attached adults may be helpful to the extent that they maintain low levels of yearning thoughts about the deceased, leading to less overt distress and non-acceptance during everyday life. However, with the growing research on implicit cognitive processing [4,5, 11, 12], the significance and direction of the avoidant attachment mediator pathway may instead underscore a risk that bereaved avoidantly attached older adults tend to actively deny and suppress their attachment-related thoughts in their cognitive attempt to downplay their attachment needs, and fail to acknowledge and resolve their implicit levels of yearning thoughts, distress and non-acceptance about the loss. By reducing their cognitive defenses in times of loss, avoidantly attached adults may achieve a more complete integration of their revised working models without the lost partner, and hence a more complete grief resolution.

### Romantic Anxious Attachment Dimension and Grief

The result offers support for the hypothesis that participants who self-report higher scores on the ECR anxious attachment dimension would experience more pining thoughts for the deceased, leading to greater distress and greater non-acceptance about the death, as self-reported by TRIG-Present. The influence of yearning thoughts is likewise indicated by the significant mediator pathways (in Figs 2 and 3) in contrast to the non-significant direct pathways between participants’ anxious attachment dimension and their levels of emotions and non-acceptance (in Fig 2).

The current data echo the unequivocal past findings [13, 14, 15, 16] that anxiously attached adults are prone to experiencing greater grief in times of bereavement. The present study, moreover, went one step further by identifying the psychological mechanism responsible for this—that a high, unremitting volume of yearning thoughts is detrimental to the ability to regulate distress and accept the painful loss.

The statistical significance and direction of the anxious attachment mediator path model is consistent with existing literature that anxiously attached adults adopt cognitive hypervigilance to negative attachment stimuli, threats and memories [4, 5, 10] and are less capable of suppressing attachment-related thoughts [7, 11], resulting in immense distress.

Overall, the mediator pathways present important implications for grief therapy. The findings suggest that participants adopt different grieving patterns according to their attachment representations. These attachment representations form the basis for their differential nature of yearning thoughts, with these thoughts critically influencing their conscious self-reported levels of emotions and acceptance about the loss. The importance of attunement between one’s cognitive and affective states for emotional well-being is supported by various therapeutic approaches for pathological grief and other emotional difficulties. These include cognitive behavioural interventions [29, 30, 31, 32], mindfulness training [33], meaning reconstruction therapy [34] and mentalization-based treatments [35]. Findings from this study may assist in the development of grief therapy according to each person’s way of relating in attachment relationships and particularly so in their organization of attachment-related thoughts. Specifically, bereaved older adults who self-identify as being more avoidantly attached can be encouraged during grief therapy to express and process more of their attachment-related thoughts about the deceased, to assist them to grieve more thoroughly. Conversely, bereaved older adults who self-identify as being more anxiously attached can be supported to reduce their yearning thoughts about the deceased, such as attending more to practical tasks and forward-looking aspects of their new life without their lost loved one, so as to deter against the development of pathologically excessive, debilitating grieving.

### Strengths and Limitations of the Study

Several limitations exist in the study. Somatic symptoms and implicit processes of grief were not assessed, comparisons with a younger sample could have been incorporated, and results from this sample of participants may not readily generalize to the wider population of grieving older adults in Australia. In addition, the study was cross-sectional in design. Cross-sectionality reduces the degree to which causal inferences can be made. Future researchers may address the problem of cross-sectionality by using longitudinal data in which the attachment dimensions are measured at Time 1, thoughts at Time 2, and non-acceptance and emotions at Time 3.

The current research provides a springboard for a richer investigation into the role of thoughts in grieving adults with different attachment representations; in particular, for the self-reported avoidantly attached older adults. We suggest future researchers consider using a self-report measure that assesses the dismissing-avoidant and fearful-avoidant attachment subtypes respectively, as well as an assessment of implicit cognitive processing and emotions of grieving. Finally, future researchers who aim to replicate this study could recruit a larger, more diverse and more representative sample of bereaved older adults in Australia.

## Supporting Information

S1 Fig Individual Participants’ Mean Scores for Avoidant Attachment, Anxious Attachment, Thoughts, Emotions, and Non-Acceptance. (SAV)
